# Case Report: Using Point-of-care Ultrasound as a Tool to Identify a Urethral Foreign Body

**DOI:** 10.5811/cpcem.2020.10.49290

**Published:** 2021-01-22

**Authors:** Jacob Frier, Elizabeth Nicholas, Paul Klawitter

**Affiliations:** SUNY Upstate Medical University, Department of Emergency Medicine, Syracuse, New York

**Keywords:** Point-of-care ultrasound, genitourinal, foreign body

## Abstract

**Introduction:**

When patients present to the emergency department with retained urethral foreign objects, imaging is crucial for identifying and further describing the object(s). Imaging is also important to plan the management and to assess the potential complications of foreign object removal. Ultrasonography is sometimes used for this purpose and can often provide more information on the object and its location and characteristics than plain radiographs.

**Case Report:**

This case report discusses the identification and characterization of a retained urethral foreign object that was not seen on plain radiography.

**Conclusion:**

While ultrasonography has its own limitations, in the cases of retained foreign objects, it can provide preferable imaging and can help guide the management of these patients.

## INTRODUCTION

Urethral foreign body (FB) is an uncommon complaint in the emergency department.^2^ Typically, these retained urethral FBs are either iatrogenic and retained from a procedure, catheter, or implant, or they are the result of autoerotic stimulation practiced by some individuals.^2^–^6^ Even more rarely is a FB placed intentionally in one’s urethra as an attempt at self-harm, and this is usually associated with a history of mental illness.^5^–^7^ Urethral FBs can have many serious complications, including urinary retention (and secondary hydronephrosis and obstructive nephropathy); infection (with the object acting as a nidus until removal); trauma to the urethra and surrounding structures; urethral scarring and subsequent stricture; and sexual dysfunction.^3^–^6^ For these reasons, timely removal of retained urethral FBs is important in preventing serious and permanent complications.

Localizing these FBs is often performed using radiographs, although ultrasound is becoming increasingly used.^1^,^6^,^8^ In many ways, ultrasound may be a preferable modality for imaging as it provides dynamic views and, if the location is superficial enough, can provide high definition images of the FB and surrounding structures.^9^–^11^ Imaging plays a vital role in the management of retained urethral FBs by helping to localize the object in relationship to other structures, and to ascertain information on the size, shape, mobility, and susceptibility to various removal techniques. In this case, we discuss a patient with a retained urethral FB that was only seen on ultrasound imaging, and which the ultrasound assisted in determining the best means of foreign object removal.

## CASE REPORT

A 29-year-old male-to-female transgender patient presented to the emergency department complaining of inability to urinate. The patient had a long history of self-injury and genital self-mutilation, including placing foreign bodies in her penile urethra. The patient stated that approximately eight hours prior to arrival, she had intentionally placed a baby carrot inside her urethral meatus and then pushed the carrot entirely into the urethra and continued to apply pressure to move the FB as proximal as she could. She reported minimal pain during the time of insertion, but since then she had gradually worsening severe suprapubic pressure and penile pressure that radiated to the scrotum. She had not been able to void since inserting the FB. Exam showed slight erythema and irritation of penile meatus, no palpable mass within the penis, non-tender testicles, and a palpable, firm, cylindrical object in the anterior perineal area when palpated through the scrotum. In addition, patient had a tender and distended bladder on suprapubic palpation.

**Image 1:**
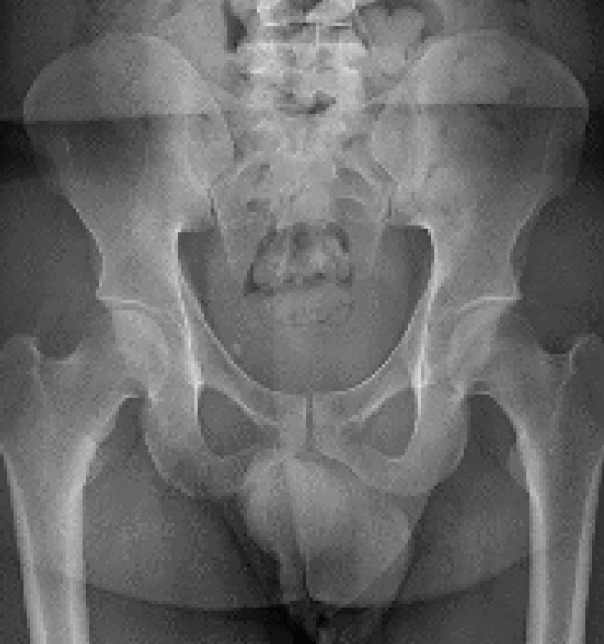
A plain anteroposterior view of the patient’s pelvis, not showing any foreign body.

AP and lateral radiographs were obtained of the pelvis, but no obvious foreign object or mass was clearly visualized (Image 1).

**Image 2:**
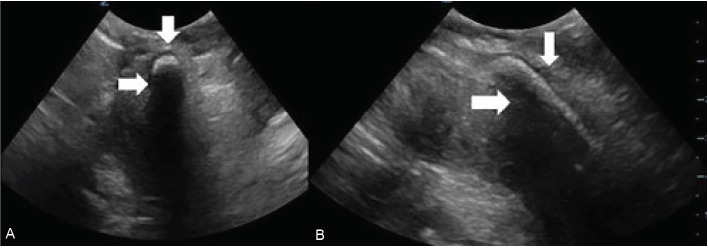
(A) Point-of-care transverse image of the urethra was obtained trans-scrotally. A hyperechoic foreign body (vertical arrow) with shadowing (horizontal arrow) within the penile urethra was identified. (B) Point-of-care ultrasound of the urethra in long axis was obtained trans-scrotally. A hyperechoic foreign body (vertical arrow) with shadowing (horizontal arrow) within the penile urethra was identified.

Point-of-care ultrasound was used for further evaluation. Multiple transverse and longitudinal views were obtained directly through the penis, but again, no foreign objects or other obvious abnormalities were identified. A trans-scrotal approach was made with the ultrasound probe, focusing on the area with the palpable cylindrical mass. Using this view, a distinct cylindrical mass was identified in both transverse and long axis (Image 2).

**CPC-EM Capsule**

What do we already know about this clinical entity?

Many foreign bodies (FB) are radiolucent, making traditional radiography unhelpful. Point-of-care ultrasound (POCUS) has become a useful tool in FB identification.

What makes this presentation of disease reportable?

This is a unique case of a urethral FB not seen on plain radiograph that was easily visualized with POCUS. Ultrasound was also used to plan FB removal.

What is the major learning point?

When urethral foreign body is suspected, POCUS may be a fast and easily accessible tool to aid in diagnosis and removal planning.

How might this improve emergency medicine practice?

Using POCUS for radiolucent urethral FBs may save time to both diagnosis and removal when compared to ureteroscopy and/or computed tomography.

**Image 3:**
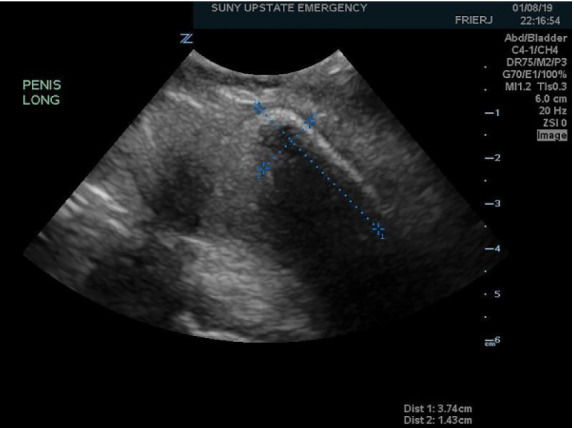
Urethral foreign body measuring 3.74 × 1.43 centimeters, as measured in long axis on point-of-care ultrasound.

Palpation of the mass while obtaining the ultrasound showed that it was mobile and appeared to be within the urethra, distal to the prostate. The object was measured (Image 3). The bladder appeared distended, but otherwise no foreign objects were noted in the bladder on ultrasound.

Urology was consulted, and based on ultrasonographic localization, was able to palpate the proximal tip of the mass. The urology resident was then able to hook a fingertip around the proximal tip and push the foreign object distally through the urethra until it was palpable in the penis. External manipulation was continued, and the urology resident was able to “milk” the foreign object entirely out of the urethra. An approximately 5- centimeter baby carrot was removed fully intact, and the patient was able to urinate immediately afterward with significant improvement in pain. The patient had no obvious complications, and was discharged on ciprofloxacin for urinary tract infection prophylaxis.

## DISCUSSION

When retained urethral foreign objects are suspected, imaging is important for diagnosis and prediction of possible complications, as well as for planning the removal of the foreign object and further management. Ultrasound has been shown to be an effective tool in confirming the presence of, locating, and determining the characteristics of a urethral foreign object. In this case, plain radiographs were not able to visualize a retained urethral foreign object, while ultrasound confirmed its presence. POCUS also demonstrated mobility of the object and its shape, which indicated that external manipulation could be a viable means of removal. Because of this, more invasive measures, such as urethroscopy, and any associated potential harms were avoided.

The limitations of POCUS can include missed foreign object depending on the depth, location, composition, and patient tolerance. Likewise, radiographs are often helpful in determining the exact size and shape of the object, and can highlight metal vs non-metal structures, but ultrasound is limited in this aspect due to various potential forms of artifact, especially with objects that ultrasound waves do not penetrate well. However, despite these limitations, as demonstrated in this case, POCUS can still be a useful and sometimes crucial tool in the management of retained urethral foreign objects.

## CONCLUSION

Point-of-care-ultrasound can be a useful tool to evaluate for urethral foreign bodies. It is fast and effective, and location can sometimes be immediately determined. In this case, the foreign body could not be identified with plain radiography. Here we show an example where POCUS proved to be diagnostic in the identification of a urethral foreign body.

## References

[1] Avery LL, Scheinfeld MH (2013). Imaging of penile and scrotal emergencies. Radiographics.

[2] Odoemene CA, Onuh CA (2017). Foreign bodies in the urinary bladder - case series. J West Afr Coll Surg.

[3] Stamatiou K, Moschouris H (2016). A rubber tube in the bladder as a complication of autoerotic stimulation of the urethra. Arch Ital Urol Androl.

[4] Unruh BT, Nejad SH, Stern TW (2012). Insertion of foreign bodies (polyembolokoilamania): underpinnings and management strategies. Prim Care Companion CNS Disord.

[5] Rahman NU, Elliott SP, McAninch JW (2004). Self-inflicted male urethral foreign body insertion: endoscopic management and complications. BJU Int.

[6] Barzilai M, Cohen I, Stein A (2000). Sonographic detection of a foreign body in the urethra and urinary bladder. Urol Int.

[7] Palmer CJ, Houlihan M, Psutka SP (2016). Urethral foreign bodies: clinical presentation and management. Urology.

[8] Shokoohi H, Kendrick Z, Sikka N (2018). Sonographic localization of a retained urethral foreign body in an elderly patient. J Clin Ultrasound.

[9] Bray PW, Mahoney JL, Campbell JP (1995). Sensitivity and specificity of ultrasound in the diagnosis of foreign bodies in the hand. J Hand Surg Am.

[10] Schlager D, Sanders AB, Wiggins D (1991). Ultrasound for the detection of foreign bodies. Ann Emerg Med.

[11] Graham DD (2002). Ultrasound in the emergency department: detection of wooden foreign bodies in the soft tissues. J Emerg Med.

